# A comparison of plotless density estimators using Monte Carlo simulation on totally enumerated field data sets

**DOI:** 10.1186/1472-6785-8-6

**Published:** 2008-04-17

**Authors:** Neil A White, Richard M Engeman, Robert T Sugihara, Heather W Krupa

**Affiliations:** 1 Department of Primary Industries and Fisheries and Agricultural Production Systems Research Unit, Toowoomba, Queensland, Australia; 2 National Wildlife Research Center, 4101 Laporte Ave, Fort Collins, CO, USA; 3 National Wildlife Research Center, USDA/APHIS/ADC, Hawaii Field Station, P.O. Box 10880, Hilo, Hawaii 96721, USA

## Abstract

**Background:**

Plotless density estimators are those that are based on distance measures rather than counts per unit area (quadrats or plots) to estimate the density of some usually stationary event, e.g. burrow openings, damage to plant stems, etc. These estimators typically use distance measures between events and from random points to events to derive an estimate of density. The error and bias of these estimators for the various spatial patterns found in nature have been examined using simulated populations only. In this study we investigated eight plotless density estimators to determine which were robust across a wide range of data sets from fully mapped field sites. They covered a wide range of situations including animal damage to rice and corn, nest locations, active rodent burrows and distribution of plants. Monte Carlo simulations were applied to sample the data sets, and in all cases the error of the estimate (measured as relative root mean square error) was reduced with increasing sample size. The method of calculation and ease of use in the field were also used to judge the usefulness of the estimator. Estimators were evaluated in their original published forms, although the variable area transect (VAT) and ordered distance methods have been the subjects of optimization studies.

**Results:**

An estimator that was a compound of three basic distance estimators was found to be robust across all spatial patterns for sample sizes of 25 or greater. The same field methodology can be used either with the basic distance formula or the formula used with the Kendall-Moran estimator in which case a reduction in error may be gained for sample sizes less than 25, however, there is no improvement for larger sample sizes. The variable area transect (VAT) method performed moderately well, is easy to use in the field, and its calculations easy to undertake.

**Conclusion:**

Plotless density estimators can provide an estimate of density in situations where it would not be practical to layout a plot or quadrat and can in many cases reduce the workload in the field.

## Background

Plotless density estimators are those that based on distance measures rather than counts per unit area (quadrats or plots) to estimate the density of some fixed event, e.g. burrow openings, damage to plant stems, etc. Plotless density estimators can provide an estimate of density in situations where it would not be practical to layout a plot or quadrat, e.g. difficult terrain, crops, situations where a low impact is required. These techniques make certain assumptions about the spatial distribution of the event that in the worst case assume that the event is randomly distributed, a situation that occurs infrequently in nature. Other techniques permit greater degrees of non-randomness. It is important therefore to understand when a certain plotless density estimator is robust to departures from non-randomness.

An evaluation of which plotless density estimator (PDE) is suitable for a given field situation requires examination of fully enumerated field populations and is ideally suited to computer simulation. Inferences about PDEs using simulated populations [1] are limited because field data rarely consists of a single type of spatial pattern. Instead natural populations tend to occur as a mixture of spatial patterns at various levels of intensity and grain (intensity is the variability in pattern seen from place to place and grain is an expression of the amount of spacing between them, [2]). Some plotless density estimators are better at handling departures from randomness due to the intensity and grain of the overall spatial pattern.

## Methods

### Estimation Methods Used

We selected the eight best estimators from the 24 evaluated by [1] to test using seventeen fully enumerated field data sets. In the discussion that follows the closest individual (CI) is the individual that is closest to the random sample point and this individual can have a nearest neighbor (NN). The closest individual to the NN is referred to as the second nearest neighbor (2NN). One or more of the following distances need to be measured depending on the estimator: from the i^*th *^random point to the first, second or third closest individual; from the closest individual to the first or second nearest neighbor and; the distance from a transect baseline of width w, to the g^*th *^event such that all g events are within the transect. Estimators used in this study (Table 1) comprise four general types: basic distance; Kendall-Moran; ordered distance and angle order; and variable area transect. The quadrat method was done to check that the simulation routines were working correctly (see Additional file 1) and not as an explicit test of this method as this has been done elsewhere [1,3]. No attempt was made to optimize the dimensions of the quadrat or the VAT. The latter has been dealt with explicitly elsewhere [4].

**Table 1:** Summary of estimators used, their formulae and main reference.

Estimator	Formula^§^	Reference
**Basic Distance (BD) estimators**		
Compound of CI, NN & 2NN (BDAV3)	*BDCI *= 1/(4 [∑ *R*_(1)*i*_/*N*]^2^)	[1]
	*BDNN *= 1/(2.778 [∑ *H*_(1)*i*_/*N*]^2^	
	*BD*2*N *= 1/(2.778 [∑ *H*_(2)*i*_/*N*]^2^	
	BDAV3 = (BDCI + BDNN + BD2N)/3	
**Kendall-Moran (KM) estimators**		
CI and NN Search areas pooled (KMP)	*KMP *= {[∑ (*p*_*i *_+ *n*_*i*_)] - 1}/∑ *B*_*i*_	[5,6]
CI, NN and 2NN search areas pooled (KM2P)	*KM*2*P *= {[∑ (*p*_*i *_+ *n*_*i *_+ *m*_*i*_)] - 1}/∑ *C*_*i*_	[5]
**Ordered Distance (OD) estimators**		
Second Closest Individual (OD2C)	*OD*2*C *= (2*N *- 1)/*π*∑ (*R*_(2)*i*_)^2^	[7,8]
Third closest Individual (OD3C)	*OD3C *= (3*N *- 1)/*π*∑ (*R*_(3)*i*_)^2^	
**Angle-Order (AO) estimators**		
Second closest individual in each quadrant (AO2Q)	$AO2Q=28N/\pi \sum 1/R_{(2)ij}^{2}$	[7,8]
Third closest individual in each quadrant (AO3Q)	$AO3Q=44N/\pi \sum 1/R_{(3)ij}^{2}$	[7,8]
**Variable Area Transect (VAT)**		
Variable Area Transect	*V AT *= (3*N *- 1)/(*w*∑ *l*_*i*_)	[9]
**Quadrat (QUAD)**		
Quadrat	*QUAD *= ∑ *q*_*i*_/*l*_*i*_*w*_*i*_*N*)	[17]

CI – closest individual, NN – nearest neighbor, 2NN – second nearest neighbor. R_(1)*i *_= the distance from the i^*th *^sample point to the CI; R_(2)*i *_= the distance from the i^*th *^sample point to the second CI; R_(3)*i *_= the distance from the i^*th *^sample point to the third CI; R_(3)*ij *_= the distance from the i^*th *^sample point to the third CI for the j^*th *^quadrant; H_(1)*i *_= the distance from the ith CI to its NN; H_(2)*i *_= the distance from the NN at the i^*th *^random point; p_*i*_, n_*i*_, m_*i *_= the number of Cis, NNs and 2^*nd *^NNs respectively, B_*i *_= the total search area at the i^*th *^sample point for the CI and NN combined; C_*i*_, = the total search area at the i^*th *^sample point for the CI, its NN, and the second NN combine; N = the sample size (number of random sample points used to gather distance information); w_*i *_= width of quadrat; w = width of transect; l_*i *_= length of quadrat.

Basic distance estimators assume a random spatial pattern and the measurements taken are similar to those used for deriving indices of aggregation [2]. Only one basic distance estimator is considered in this paper. It is the average of three basic distance estimators that measure the distance to the closest individual, the nearest neighbor and the second nearest neighbor [1].

**Figure 1:**
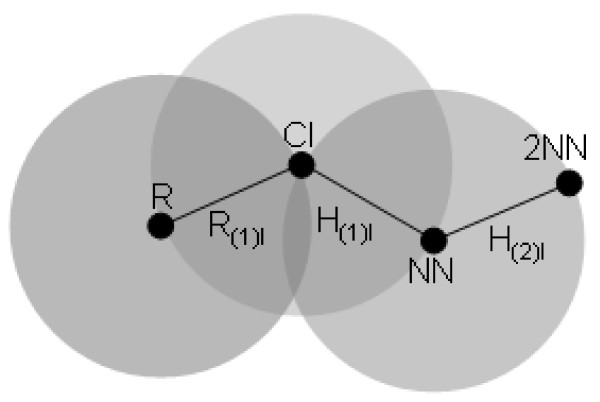
**Schematic representation of how KM2P and BDAV3 are implemented in the field**. Shading shows the search area less intersection used in the calculation of KM2P. R – the random sample point CI – closest individual; NN – nearest neighbor; 2NN – second nearest neighbor, R_(1)*i *_= the distance from the i^*th *^sample point to the CI; H_(1)*i *_= the distance from the i^*th *^CI to its NN; H_(2)*i *_= the distance from the NN at the i^*th *^random point.

Kendall-Moran estimators, [5,6] although relatively simple to implement in the field, these methods present calculation difficulties in order to derive the density estimate. Calculations are complicated because the estimator uses combined search areas, i.e. the area that must be traversed to locate the required individual, minus their intersection (Figure 1). While this is difficult enough for the closest individual and the nearest neighbor search area it becomes a great deal more difficult when the second nearest neighbor search area is also considered. An algorithm for its calculation was originally developed for the simulations by [1], and was incorporated into the simulation programs used here.

Ordered distance and angle order methods[7,8] are very similar. Both utilize distance to the closest individual. Angle order methods use measurements within each of a specified number of sectors surrounding the random sampling point while ordered distance methods use the whole search area around the sampling point. Angle order methods are less effected by non-randomness in a clumped population if the events are essentially random within each sector. Both types of estimator can be extended to use more than the first closest individual and in angle-order methods these measurements are repeated for each sector.

The variable area transect method uses a fixed width, variable length transect that is extended until the g^*th *^individual is encountered. In this study we used g = 3. A random distribution of events is assumed since the method relies on density being a function of transect length. [9] suggests that pre-sampling should be undertaken to ensure that homogenous strata could be defined, although [1] found it to be fairly robust. This method is easy to use in the field as the user needs only to search a strip transect in one direction. Transect width is the most important factor affecting estimation quality [10]. Transect width was set at 2 m to avoid comparisons becoming difficult between optimised and unoptimised estimators.

### Simulation Study Design and Data Sets

Eight plotless density estimators were examined in the present study using 5000 Monte Carlo simulations, Table 1. The simulation program was written in Fortran 77, and each simulation was a specific combination of a spatial data set and sample size (10, 25, 50 and 100 samples per simulation were undertaken). The uniform random number generator, UNIF [11], was used to locate sampling points and, where required, the VNORM routine [11] was used to convert uniform random numbers to normal random numbers to generate the synthetic data sets used for comparison with natural data sets (see below). The input for each simulation included: the name of the data file containing the location of all events as X-Y coordinates on a Cartesian plane; selects the number of samples to be taken; the sizes of the VAT width and quadrat; an output file specification and; the number of simulations to be performed. These inputs were provided within in a batch-processing environment and could be left to run unattended. The output file, one for each data set comprised the estimated density, relative bias and relative root mean square error for each estimator.

### Natural data sets

Seventeen data sets (Table 2) were obtained from unpublished studies by the authors and colleagues that included animal damage to rice and corn, bird nest locations, active rodent burrows and distribution of plants. Densities ranged from 0.06 m^-2 ^(bee-eater nest sites) to 19.3 m^-2 ^(damaged sugar). A boundary strip of 10% of the length and width of the extent of the population of points was used to remove the bias associated with sampling close to the edge of the study area.

**Table 2:** Description of data sets used and density of the event.

Data Set	Description	n	Dimensions (m)	Density (m^-2^)
Bee eater	Bee eater nest sites	64	41.5*24	0.06
Corn 1	Rat damage to corn in the Philippines for three different fields	2406	89.25*103.2	0.26
Corn 2		1596	86.25*121.6	0.15
Corn 3		1342	99.2*96.75	0.14
PG 92	Active pocket gopher burrows – 1992	132	28.5*22	0.21
PG 93	Active pocket gopher burrows – 1993	136	32.6*22.5	0.19
Rice 1	Rat damage to rice in the Philippines for five different fields	1678	63.5*12.25	2.16
Rice 2		177	7.31*16.66	1.45
Rice 3		3105	17.8*19.8	8.81
Rice 4		262	18.36*8.16	1.75
Rice 5		275	21.08*7.99	1.63
Sugar 1	Rat damage in sugarcane, Mauna Kea Agribusiness fields, Hawaii, USA	921	7.99*5.96	19.34
Sugar 2		199	7.77*5.94	4.31
Sugar 3		689	7.98*5.98	14.44
Sugar 4		174	7.48*6.52	3.57
Waterfowl	Alaskan waterfowl nests	497	26.3*5.9	3.20
Xanth	Distribution of grass trees (*Xanthorrhoea *sp.), Bribie Island, Queensland, Australia	748	25*50	0.60

For ground or cliff nesting birds the density of nest sites provide important information on the number of breeding females or pairs. Two data sets were used with densities of 0.06 (bee eater) and 3.2 m^-2 ^(Alaskan waterfowl nests).

Burrowing species such as gophers and rabbits can be monitored through the presence of active burrows. Two data sets of a population of pocket gophers measured in two successive years were used to demonstrate the application of PDE as a suitable method for monitoring populations.

The use of PDEs for monitoring damage to crops was done using corn and rice in the Philippines, and sugar cane in Hawaii.

The remaining data set is from a coastal sand island, north of Brisbane, Australia. Grass trees, *Xanthorrhoea *sp., grow in heath communities inland from the foredunes. Unlike the crop data sets these are naturally occurring communities.

### Simulated data sets

Five data sets whose spatial characteristics were predetermined were also included for comparison. The artificial data sets (where n is the number of individuals, *λ *is the density m^-2^) had distributions that were Poisson (n = 100, *λ *= 1), uniform – regular lattice (n = 100, *λ *= 1), hexagonal – regular triangular (n = 100, *λ *= 0.9), first-order clumped (n = 100, *λ *= 1.1, number of offspring per parent (nop) = 10, clump radius (cr) = 0.5 m) and second order clumped (n = 100, *λ *= 2.1, nop = 10, cr = 0.5 m). The Poisson or random pattern was created by generating the required number of random coordinates within the designated area. The uniform data set was generated by first dividing the area into a grid of rectangles, the same number as the population size. One population member was randomly located within each grid cell. The hexagonal pattern was generated so that population members were located at the vertices of a lattice of equilateral triangles. For the clumped data sets, the required number of clump centers was randomly created within the designated area. In addition to the clump center point, offspring for the clumps were located within a designated radius from the parent. These offspring were located within the clump about the parent using coordinates randomly generated using a standard bivariate normal distribution. For the second order clumping, the individuals in the clump are used for parent points. The two individuals of the sub-clumps include the parent plus offspring points, which are randomly generated from the standard bivariate normal distribution. The radius for the sub-clump is limited to half that for the clump. The second order clumping approximates the situation that can occur with rodent damage in field crops.

### Statistics

The relative root mean square error (RRMSE) was used as the basis of comparisons between the different PDEs [1,12], where *I *is the number of simulations (5000), D_*est *_is the estimated density and *λ *is the true density in the population, such that:

$$RRMSE=\sqrt{\frac{\sum {\left(D_{est}-\lambda \right)}^{2}/\lambda^{2}}{I}}$$

In addition, relative bias (RBIAS) shows the bias relative to the true density and the direction of that bias such that:

$$RBIAS=\frac{(\sum D_{est}/I)-\lambda }{\lambda }$$

The R index, [13], was calculated for all data sets (Table 3) including examples of simulated distributions such that:

**Table 3:** R index, standard error of expected mean, s, and z statistic [13] for the data sets used. When the pattern is entirely random R = 1, if the events are uniform then R > 1 (R = 2.149 for a perfect hexagonal uniform distribution) and conversely when the population of events is clumped R < 1 (R approaches 0 for maximally clumped distribution). The z test statistic considers the null hypothesis that the spatial distribution is random. Data sets comparable to those generated in [1] in italics.

Dataset	R	s	z	Pattern
*Hexagonal*	2.15	0.003	76.53	Uniform
Rice 3	1.41	0.002	39.57	Uniform
PG 92	1.36	0.067	6.16	Uniform
Bee-eater	1.34	0.086	8.17	Uniform
PG 93	1.3	0.077	4.9	Uniform
*Uniform*	1.25	0.003	17.34	Uniform
Rice 1	1.08	0.004	6.12	Uniform
Rice 5	1.04	0.016	0.99	Random
*Poisson*	0.98	0.003	-1.1	Random
Rice 4	0.94	0.013	-1.59	Random
Xanth	0.89	0.017	-4.3	Clumped
Corn 1	0.88	0.014	-8.69	Clumped
Rice 2	0.88	0.019	-2.43	Clumped
Sugar 1	0.83	0.002	-8.15	Clumped
*Clump 1*	0.8	0.003	-14.79	Clumped
*Clump 2*	0.8	0.002	-16.34	Clumped
Sugar 3	0.76	0.003	-9.45	Clumped
Corn 2	0.75	0.039	-11.12	Clumped
Sugar 4	0.7	0.011	-6.57	Clumped
Waterfowl	0.65	0.007	-12.6	Clumped
Sugar 2	0.64	0.013	-7.32	Clumped
Corn 3	0.47	0.043	-21.97	Clumped

$$R_{O}=\frac{\sum_{i=1}^{n}r_{i}}{n}$$

where R_*O *_is the average observed nearest neighbor distance, r_*i *_is the nearest neighbor distance to the i^*th *^sample point and n is number of nearest neighbor distances measured;

$${\text{R}}_{E}=\frac{1}{2\sqrt{\lambda }}$$

where R_*E *_is the expected nearest neighbor distance for a random pattern of events;

$$\text{R}=\frac{R_{O}}{R_{E}}$$

R was calculated for the complete data set less a 10% buffer. When the pattern is entirely random R = 1, if the events are uniform then R > 1 (R = 2.149 for a perfect hexagonal uniform distribution) and conversely when the population of events is clumped R < 1 (R approaches 0 for maximally clumped distributions). The z test statistic was calculated that measured the difference between the observed and expected values of R, i.e. it considers a null hypothesis that the spatial distribution is random.

$$z=\frac{\left|R_{O}-R_{E}\right|}{se}$$

where se is the standard error of R_*E*_

$$se=\frac{0.26136}{\sqrt{n\lambda }}$$

A Spearman (rank) correlation coefficient was calculated between the log of (*λ*) and the log of D_*est *_for AO3Q, BDAV3, KM2P and VAT across all natural data sets.

## Results and Discussion

Interpretation of the performance of estimators based on relative root mean square error (RRMSE) (Table 4) and relative bias (RBIAS) (Table 5) was undertaken for estimators that were ranked highly by [1] (Table 1) for the natural and simulated data sets described in Tables 2 and 3. Complete results of the simulations are provided in Additional file 1.

**Table 4:** Mean relative root mean square error for 10, 25, 50 and 100 samples/simulation for each density estimator and each spatial pattern for the natural data sets (see Table 3)

	Sample size				Sample size			
	RRMSE				Rank			
		
Estimator	10	25	50	100	10	25	50	100

	Uniform (n = 5)							
AO2Q	0.306	0.266	0.247	0.238	7	8	8	8
AO3Q	0.280	0.247	0.232	0.224	6	7	7	7
BDAV3	0.254	0.202	0.182	0.173	3	5	5	5
KM2P	0.247	0.199	0.177	0.166	1	3	3	4
KMP	0.256	0.201	0.177	0.165	4	4	4	3
OD2C	0.307	0.229	0.202	0.188	8	6	6	6
OD3C	0.251	0.182	0.157	0.143	2	1	1	1
VAT	0.258	0.194	0.167	0.148	5	2	2	2

	Poisson (n = 2)							
AO2Q	0.392	0.353	0.341	0.333	8	8	8	8
AO3Q	0.345	0.316	0.307	0.302	7	7	7	7
BDAV3	0.270	0.157	0.114	0.091	4	3	3	3
KM2P	0.232	0.149	0.111	0.088	1	1	2	2
KMP	0.288	0.193	0.153	0.130	5	5	5	5
OD2C	0.304	0.199	0.159	0.131	6	6	6	6
OD3C	0.253	0.160	0.123	0.098	2	4	4	4
VAT	0.256	0.154	0.107	0.077	3	2	1	1

	Clumped (n = 10)							
AO2Q	0.390	0.321	0.293	0.277	2	2	3	3
AO3Q	0.362	0.307	0.284	0.271	1	1	1	2
BDAV3	0.461	0.331	0.287	0.263	6	3	2	1
KM2P	0.424	0.374	0.361	0.354	3	4	4	4
KMP	0.468	0.427	0.413	0.406	7	7	6	6
OD2C	0.491	0.466	0.459	0.455	8	8	8	8
OD3C	0.448	0.426	0.420	0.417	5	6	7	7
VAT	0.439	0.414	0.407	0.403	4	5	5	5

	All (n = 17)							
AO2Q	0.368	0.311	0.287	0.274	4	4	4	4
AO3Q	0.338	0.292	0.273	0.263	1	2	2	2
BDAV3	0.380	0.273	0.236	0.216	6	1	1	1
KM2P	0.346	0.297	0.279	0.269	2	3	3	3
KMP	0.387	0.335	0.316	0.305	7	7	7	7
OD2C	0.417	0.367	0.350	0.340	8	8	8	8
OD3C	0.369	0.325	0.311	0.301	5	6	6	6
VAT	0.366	0.321	0.303	0.291	3	5	5	5

**Table 5:** Mean relative bias for 10, 25, 50 and 100 samples/simulation for each density estimator for each spatial pattern (see Table 3)

	Sample size				Sample size			
	RBIAS				Rank			
		
Estimator	10	25	50	100	10	25	50	100

	Uniform (n = 5)							
AO2Q	0.222	0.225	0.225	0.225	8	8	8	8
AO3Q	0.205	0.207	0.207	0.207	7	7	7	7
BDAV3	-0.095	-0.136	-0.147	-0.154	4	4	5	6
KM2P	-0.136	-0.145	-0.148	-0.150	6	6	6	5
KMP	-0.130	-0.141	-0.145	-0.148	5	5	4	4
OD2C	-0.051	-0.077	-0.091	-0.097	1	1	2	2
OD3C	-0.070	-0.091	-0.101	-0.106	2	3	3	3
VAT	-0.074	-0.080	-0.081	-0.083	3	2	1	1

	Poisson (n = 2)							
AO2Q	0.324	0.324	0.326	0.325	8	8	8	8
AO3Q	0.295	0.295	0.296	0.296	7	7	7	7
BDAV3	0.073	0.014	-0.002	-0.009	6	2	2	2
KM2P	-0.037	-0.050	-0.052	-0.052	3	4	4	3
KMP	-0.070	-0.089	-0.092	-0.094	5	6	6	6
OD2C	-0.045	-0.065	-0.070	-0.071	4	5	5	5
OD3C	-0.031	-0.047	-0.051	-0.052	2	3	3	4
VAT	0.019	0.005	0.000	-0.003	1	1	1	1

	Clumped (n = 10)							
AO2Q	-0.079	-0.082	-0.081	-0.080	2	3	3	3
AO3Q	-0.063	-0.065	-0.065	-0.064	1	2	2	2
BDAV3	0.080	-0.008	-0.036	-0.049	3	1	1	1
KM2P	-0.319	-0.325	-0.333	-0.337	4	4	4	4
KMP	-0.350	-0.377	-0.386	-0.391	5	5	5	5
OD2C	-0.410	-0.435	-0.444	-0.447	8	8	8	8
OD3C	-0.376	-0.399	-0.407	-0.410	7	7	7	7
VAT	-0.365	-0.387	-0.393	-0.396	6	6	6	6

	All (n = 17)							
AO2Q	0.055	0.054	0.055	0.055	2	2	1	1
AO3Q	0.056	0.056	0.056	0.056	3	3	2	2
BDAV3	0.033	-0.039	-0.061	-0.072	1	1	3	3
KM2P	-0.227	-0.241	-0.246	-0.249	4	4	4	4
KMP	-0.254	-0.276	-0.282	-0.286	7	7	7	7
OD2C	-0.266	-0.290	-0.300	-0.304	8	8	8	8
OD3C	-0.248	-0.270	-0.278	-0.281	6	6	6	6
VAT	-0.236	-0.253	-0.257	-0.260	5	5	5	5

**Figure 2:**
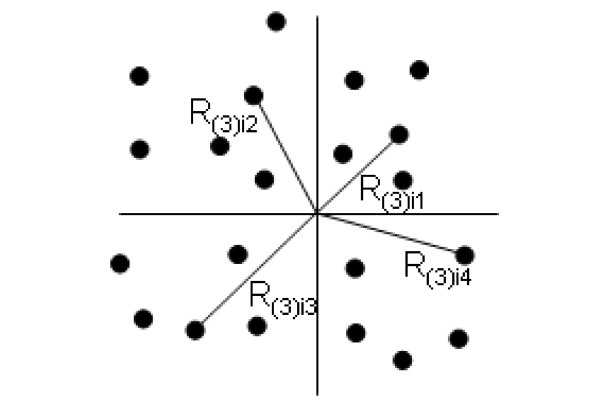
**Schematic representation of how AO3Q is implemented in the field**. The order of the quadrants is arbitrary. In practice much time is spent deciding which is the third closest individual and into which quadrant an individual lies. R_(3)*ij *_= the distance from the i^*th *^sample point to the third CI for the j^*th *^quadrant.

An ideal estimator is one that is robust across many spatial patterns, i.e. RRMSE and RBIAS are low, and where the amount of fieldwork required can be minimized or at least be undertaken efficiently. Basic distance estimators were largely dismissed by [1] because they showed poor performance for clumped data sets, however, they performed much better in this study than most other methods with the exception of the angle-order estimators (Table 4). Across all data sets the compound estimator, BDAV3 (Figure 1), was the best-ranked method for sample sizes greater than 10 and performed well in terms of bias. BDAV3 was less suited for Poisson distributions. For these distributions Kendall-Moran estimator (KM2P) was ranked first when sample size was 10 or 25. For sample sizes of 50 or 100 the variable area transect (VAT) method was ranked first. The highest ranked estimators for the clumped distribution were the two angle order estimators AO3Q (Figure 2) and AO2Q. The VAT performed moderately well overall and is far easier to implement in many situations.

Absolute relative bias (i.e. regardless of sign) for the AO and BD estimators was an order of magnitude smaller than the others for clumped data sets. However, AO estimators showed higher positive bias for Poisson data sets compared to the near zero for the others. In uniform data sets the OD and VAT estimators showed a RBIAS close to zero.

BDAV3 and KM2P use the same field methodology, however, data processing is much simpler for BD than for KM estimators. These estimators use information from the closest individual, distance to its nearest neighbor and the second nearest neighbor and that may help to explain why they are robust across all spatial patterns studied here, compared to estimators such as AO that rely on information derived from the closest individual.

Whereas the calculation for KM2P looks deceptively simple (Table 1, Figure 1), delineating search areas has to be done algorithmically when the number of samples is realistically large and this difficulty needs to be considered beforehand. The KM calculation is suggested when the distribution is likely to be uniform. The formulae AO3Q is simple to undertake and the methods are suited to situations where movement and/or vision is good, e.g. it may not be suitable for crops where excessive movement would cause damage. The estimator with the lowest RRMSE for each data type for a sample size of 50 was: uniform – OD3C, poisson – VAT, clumped – AO3Q, overall – BDAV3.

For uniform patterns the OD3C, VAT or KM2P methods were the most suitable, however, the method of searching in VAT is the simplest to implement. The fieldwork required for BDAV3 and KM2P are the same and although BDAV3 is much easier to calculate it is less able to cope with uniform data sets. The selection of the required sample size should be undertaken on a case-by-case basis using a pilot study. Accuracy will be improved with larger sample sizes and the techniques used to minimize the variance through stratified sampling, randomization, etc. should be employed.

**Figure 3:**
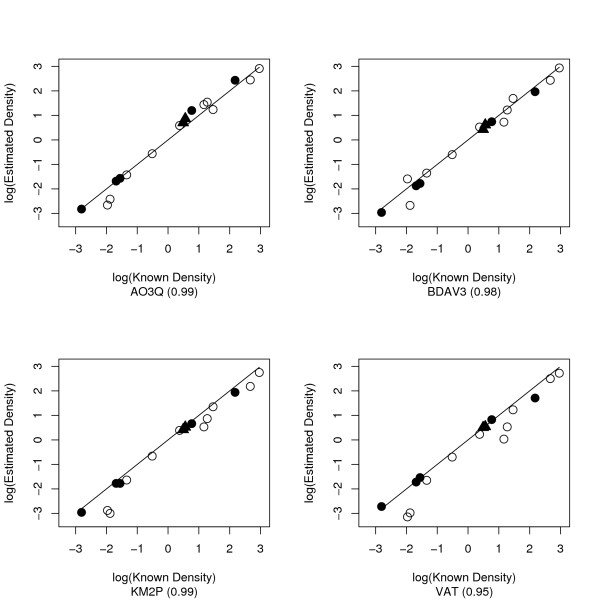
**Correlation between mean density estimate against known density for all data sets**. Line shows complete agreement between known and estimated density. Spearman's correlation coefficient shown in parentheses. Symbols denote spatial pattern of data set: Uniform – filled circle, Poisson – filled triangle, Clumped – open circle.

The VAT method would seem the most straightforward to utilize in most field situations, and under optimized sampling constraints the method holds promise for row crops [14]. In comparisons between the known density and the mean estimated density (Figure 3), the VAT had the lowest correlation coefficient of the four estimators tested in this way, although this was still 0.95. This suggests that ranking solely on RRMSE might lead one to favor methods that are difficult to implement in the field.

Furthermore, the present study aimed to examine PDE methods as originally presented, without attempting to improve performance through optimizing procedures. Thus we examined VAT sampling using g = 3. The number of individuals for which to search has been optimized with substantial improvements in estimation quality for g ≥ 5 [4,10,14]. Other than the KM2P estimator, most other PDE forms hold opportunity for improving estimation by optimizing the number of population members for which to search. [15] examined this for ordered distance estimation using simulated data sets similar to the approach taken by [4]. Angle-order methods could be optimized for the number of individuals to search in each sector, and the number of sectors into which the search area around the random sampling point is divided.

**Figure 4:**
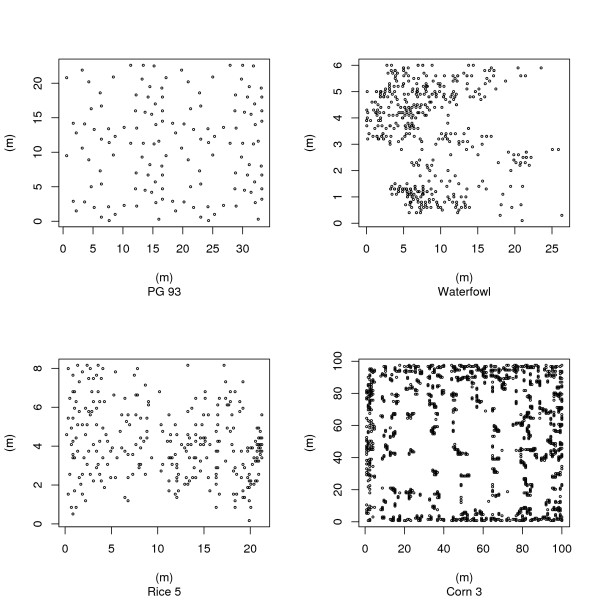
**Examples of diversity of spatial patterns found**. (a) uniform distribution of pocket gopher burrows; (b) aggregated nesting pattern of waterfowl; (c) random pattern of rodent damage in rice; (d) highly clumped damage within a cornfield.

When damage is the event to be estimated and is caused by an animal that invades a crop or forestry coup it is usual to find the damage along the edge. Figures 4a-d show the diversity of spatial patterns exhibited in the data sets. Figure 4a shows the distribution of pocket gopher burrows with a uniform distribution, while Figure 4b shows an aggregated nesting pattern of waterfowl. Figure 4c shows a random pattern of rodent damage in rice while 4d is highly clumped damage within a cornfield.

**Figure 5:**
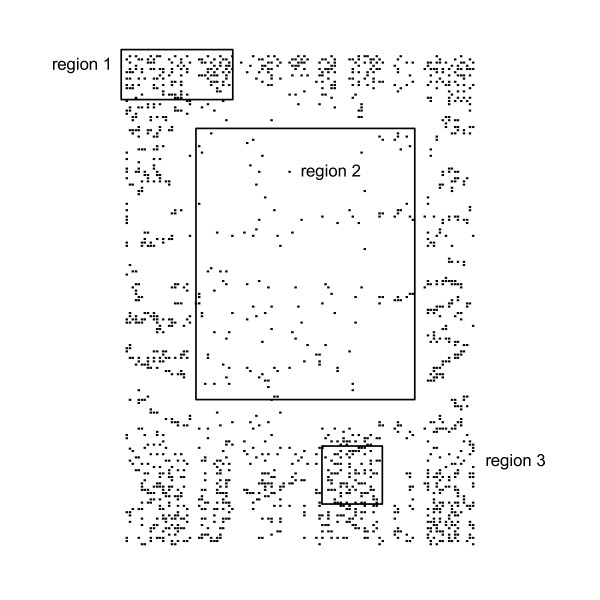
Subsets within the highly clumped Corn 2 data set showing random and uniform patterns, see Table 6.

Typically the data sets of damage were clumped, however, random and uniform patterns were also found for data sets that mapped the distribution of burrows or nest sites. It is a characteristic of field data that the spatial pattern can vary within the study area. This was demonstrated by recalculating the R index for regions within the Corn 2 data set (Figure 5, Table 6). It is therefore advisable to undertake an investigation of the spatial pattern present and this can be done using either the [13] R index or the [16] Hopkins and Skellam index as part of any preliminary study using blocking to detect regions of clumping as it is this spatial pattern that causes the greatest problems with many estimators. The latter index is probably more applicable for field studies as it does not require an estimate of density beforehand. Where clumping is present angle order methods should be used.

**Table 6:** R index, standard error of expected mean, s, and z statistic [13] for subsets within Corn 2 see Figure 5.

Dataset	R	s	z	Pattern
Region 1	1.1	0.053	1.5	Random
Region 2	0.92	0.173	-1.23	Random
Region 3	1.21	0.047	3.21	Uniform

## Conclusion

Plotless density estimators can provide an estimate of density in situations where it would not be practical to layout a plot or quadrat and can in many cases reduce the workload in the field.

## Authors' contributions

NAW ran the simulations and with RME and HWK drafted and finalised the manuscript. RTS developed the original fortran code. All authors read and approved the final manuscript.

## Supplementary Material

Additional file 1 Complete results from all simulations. Click here for file
