# Supplementary material for: A comparison of plotless density estimators using Monte Carlo simulation on totally enumerated field data sets
Source: BMC Ecol. 2008 Apr 17;8:6. doi: 10.1186/1472-6785-8-6 (PMC2422836; doi:10.1186/1472-6785-8-6)
Supplement: Additional file 1 — Complete results from all simulations. [file 1472-6785-8-6-S1.pdf]

Table 1: Complete results for all estimators and data sets

				Corn 1					Corn 2		
Estimator		10	25	50	100	Mean	10	25	50	100	Mean
AO2Q	RRMSE	0.28	0.169	0.119	0.084	0.163	0.521	0.543	0.55	0.556	0.542
	RBIAS	0.077	0.013	-0.011	-0.021	0.014	-0.486	-0.534	-0.546	-0.554	-0.53
AO3Q	RRMSE	0.423	0.385	0.379	0.378	0.391	0.718	0.728	0.732	0.734	0.728
	RBIAS	-0.294	-0.338	-0.357	-0.367	-0.339	-0.706	-0.725	-0.73	-0.733	-0.724
BDAV3	RRMSE	0.264	0.18	0.137	0.113	0.174	0.406	0.329	0.304	0.292	0.333
	RBIAS	0.079	0.082	0.08	0.08	0.08	-0.276	-0.271	-0.273	-0.276	-0.274
KM2P	RRMSE	0.64	0.405	0.292	0.21	0.387	0.771	0.556	0.461	0.406	0.548
	RBIAS	0.039	0.047	0.046	0.047	0.045	-0.343	-0.333	-0.344	-0.345	-0.341
KMP	RRMSE	0.329	0.292	0.281	0.276	0.294	0.663	0.665	0.667	0.668	0.666
	RBIAS	-0.23	-0.254	-0.264	-0.268	-0.254	-0.655	-0.663	-0.666	-0.668	-0.663
OD2C	RRMSE	0.462	0.45	0.445	0.446	0.451	0.741	0.751	0.755	0.756	0.751
	RBIAS	-0.366	-0.42	-0.43	-0.439	-0.414	-0.731	-0.748	-0.754	-0.756	-0.747
OD3C	RRMSE	0.403	0.384	0.376	0.376	0.385	0.711	0.72	0.724	0.726	0.72
	RBIAS	-0.3	-0.349	-0.358	-0.367	-0.343	-0.702	-0.718	-0.723	-0.725	-0.717
QUAD	RRMSE	0.266	0.194	0.16	0.14	0.19	0.492	0.448	0.432	0.424	0.449
	RBIAS	-0.112	-0.117	-0.116	-0.116	-0.115	-0.414	-0.416	-0.416	-0.416	-0.415
VAT	RRMSE	0.229	0.16	0.127	0.107	0.156	0.477	0.44	0.427	0.42	0.441
	RBIAS	-0.077	-0.082	-0.082	-0.081	-0.081	-0.411	-0.413	-0.414	-0.413	-0.413
				Corn 3					Sugar 1		
Estimator		10	25	50	100	Mean	10	25	50	100	Mean
AO2Q	RRMSE	0.798	0.589	0.503	0.456	0.587	0.322	0.179	0.124	0.094	0.18
	RBIAS	0.646	0.506	0.456	0.431	0.51	0.073	-0.004	-0.026	-0.041	0
AO3Q	RRMSE	0.684	0.692	0.697	0.697	0.693	0.349	0.287	0.266	0.261	0.291
	RBIAS	-0.668	-0.687	-0.695	-0.696	-0.686	-0.203	-0.235	-0.239	-0.248	-0.231
BDAV3	RRMSE	0.397	0.254	0.18	0.13	0.24		0.345	0.275	0.228	
	RBIAS	-0.01	-0.006	-0.003	-0.004	-0.006		0.18	0.18	0.174	
KM2P	RRMSE	0.89	0.595	0.47	0.382	0.584	0.169	0.111	0.087	0.072	0.11
	RBIAS	-0.231	-0.257	-0.267	-0.276	-0.258	-0.053	-0.049	-0.049	-0.051	-0.051
KMP	RRMSE	0.678	0.689	0.693	0.694	0.689	0.336	0.277	0.253	0.242	0.277
	RBIAS	-0.661	-0.684	-0.691	-0.693	-0.682	-0.148	-0.199	-0.214	-0.223	-0.196
OD2C	RRMSE	0.72	0.727	0.73	0.733	0.728	0.387	0.349	0.338	0.332	0.352
	RBIAS	-0.709	-0.724	-0.729	-0.733	-0.724	-0.278	-0.313	-0.32	-0.323	-0.308
OD3C	RRMSE	0.652	0.658	0.661	0.664	0.659	0.34	0.304	0.293	0.286	0.306
	RBIAS	-0.638	-0.654	-0.659	-0.663	-0.653	-0.236	-0.269	-0.276	-0.278	-0.265
QUAD	RRMSE	0.568	0.55	0.543	0.538	0.55	0.259	0.171	0.124	0.095	0.162
	RBIAS	-0.538	-0.538	-0.537	-0.535	-0.537	-0.047	-0.048	-0.051	-0.048	-0.049
VAT	RRMSE	0.522	0.51	0.503	0.501	0.509	0.209	0.139	0.102	0.08	0.133
	RBIAS	-0.499	-0.5	-0.499	-0.499	-0.499	-0.045	-0.046	-0.049	-0.047	-0.047
				Sugar 2					Sugar 3		
Estimator		10	25	50	100	Mean	10	25	50	100	Mean
AO2Q	RRMSE	0.55	0.364	0.301	0.269	0.371	0.319	0.25	0.24	0.237	0.262
	RBIAS	0.376	0.29	0.26	0.248	0.293	-0.101	-0.185	-0.212	-0.224	-0.18
AO3Q	RRMSE	0.274	0.17	0.13	0.101	0.169	0.452	0.436	0.435	0.434	0.439
	RBIAS	-0.049	-0.058	-0.064	-0.064	-0.059	-0.383	-0.413	-0.425	-0.429	-0.412
BDAV3	RRMSE	0.649	0.437	0.343	0.274	0.426	0.53	0.331	0.241	0.175	0.319
	RBIAS	0.206	0.207	0.206	0.199	0.204	0.066	0.063	0.066	0.067	0.066
KM2P	RRMSE	0.249	0.208	0.194	0.185	0.209	0.181	0.122	0.094	0.077	0.118
	RBIAS	-0.176	-0.175	-0.177	-0.176	-0.176	-0.052	-0.053	-0.053	-0.053	-0.053
KMP	RRMSE	0.271	0.232	0.223	0.216	0.236	0.228	0.187	0.172	0.166	0.188
	RBIAS	-0.201	-0.205	-0.209	-0.209	-0.206	-0.153	-0.158	-0.157	-0.158	-0.157
OD2C	RRMSE	0.306	0.249	0.232	0.225	0.253	0.467	0.455	0.456	0.457	0.459
	RBIAS	-0.206	-0.207	-0.214	-0.215	-0.21	-0.402	-0.435	-0.447	-0.452	-0.434
OD3C	RRMSE	0.282	0.243	0.233	0.228	0.246	0.427	0.416	0.417	0.417	0.419
	RBIAS	-0.213	-0.217	-0.22	-0.221	-0.218	-0.373	-0.398	-0.409	-0.414	-0.398
QUAD	RRMSE	0.269	0.235	0.222	0.216	0.235	0.315	0.249	0.226	0.211	0.25
	RBIAS	-0.208	-0.21	-0.209	-0.209	-0.209	-0.195	-0.195	-0.197	-0.196	-0.196
VAT	RRMSE	0.235	0.216	0.208	0.205	0.216	0.287	0.238	0.221	0.211	0.239
	RBIAS	-0.203	-0.203	-0.201	-0.201	-0.202	-0.202	-0.201	-0.201	-0.201	-0.201

Table 2: Complete results for all estimators and data sets continued

				Sugar 4					Rice 1		
Estimator		10	25	50	100	Mean	10	25	50	100	Mean
AO2Q	RRMSE	0.591	0.272	0.184	0.138	0.296	0.306	0.181	0.125	0.094	0.176
	RBIAS	0.16	-0.007	-0.05	-0.072	0.008	0.056	-0.01	-0.027	-0.039	-0.005
AO3Q	RRMSE	0.42	0.375	0.366	0.364	0.381	0.373	0.275	0.235	0.22	0.276
	RBIAS	-0.307	-0.343	-0.351	-0.357	-0.34	-0.131	-0.174	-0.188	-0.196	-0.172
BDAV3	RRMSE	1.228	0.974	0.87	0.832	0.976	0.34	0.267	0.238	0.219	0.266
	RBIAS	0.757	0.756	0.756	0.77	0.76	0.2	0.198	0.202	0.2	0.2
KM2P	RRMSE	0.357	0.221	0.16	0.113	0.213	0.519	0.423	0.38	0.361	0.421
	RBIAS	0.006	0.011	0.009	0.009	0.009	0.33	0.339	0.335	0.338	0.336
KMP	RRMSE	0.531	0.527	0.529	0.53	0.529	0.363	0.215	0.154	0.11	0.21
	RBIAS	-0.478	-0.515	-0.523	-0.527	-0.511	0.105	0.068	0.06	0.052	0.071
OD2C	RRMSE	0.445	0.414	0.411	0.408	0.419	0.404	0.305	0.279	0.266	0.314
	RBIAS	-0.372	-0.392	-0.401	-0.403	-0.392	-0.164	-0.223	-0.239	-0.248	-0.218
OD3C	RRMSE	0.416	0.387	0.386	0.383	0.393	0.356	0.261	0.234	0.22	0.268
	RBIAS	-0.346	-0.366	-0.376	-0.378	-0.366	-0.13	-0.179	-0.192	-0.201	-0.175
QUAD	RRMSE	0.461	0.361	0.316	0.294	0.358	0.674	0.621	0.596	0.586	0.619
	RBIAS	0.267	0.266	0.267	0.268	0.267	0.57	0.576	0.573	0.575	0.573
VAT	RRMSE	0.473	0.387	0.347	0.331	0.385	0.625	0.579	0.557	0.551	0.578
	RBIAS	0.309	0.313	0.308	0.31	0.31	0.537	0.541	0.539	0.541	0.54
				Rice 2					Rice 3		
Estimator		10	25	50	100	Mean	10	25	50	100	Mean
AO2Q	RRMSE	0.41	0.252	0.199	0.17	0.258	0.273	0.226	0.214	0.21	0.231
	RBIAS	0.271	0.188	0.164	0.151	0.194	-0.123	-0.175	-0.19	-0.199	-0.172
AO3Q	RRMSE	0.33	0.205	0.153	0.116	0.201	0.357	0.325	0.308	0.3	0.323
	RBIAS	-0.01	-0.042	-0.052	-0.059	-0.041	-0.208	-0.251	-0.269	-0.279	-0.252
BDAV3	RRMSE	0.509	0.381	0.324	0.295	0.377	0.245	0.231	0.224	0.221	0.23
	RBIAS	0.261	0.258	0.257	0.26	0.259	-0.221	-0.22	-0.219	-0.219	-0.22
KM2P	RRMSE	0.314	0.2	0.141	0.102	0.189	0.299	0.244	0.221	0.211	0.244
	RBIAS	0.025	0.028	0.029	0.031	0.028	0.194	0.197	0.198	0.198	0.197
KMP	RRMSE	0.248	0.185	0.164	0.151	0.187	0.393	0.382	0.379	0.377	0.383
	RBIAS	-0.112	-0.132	-0.136	-0.137	-0.129	-0.363	-0.37	-0.373	-0.375	-0.37
OD2C	RRMSE	0.305	0.211	0.168	0.145	0.207	0.429	0.334	0.304	0.29	0.339
	RBIAS	-0.072	-0.101	-0.109	-0.112	-0.099	-0.118	-0.192	-0.235	-0.254	-0.2
OD3C	RRMSE	0.256	0.174	0.14	0.119	0.172	0.373	0.288	0.26	0.245	0.291
	RBIAS	-0.062	-0.086	-0.09	-0.092	-0.082	-0.111	-0.17	-0.201	-0.214	-0.174
QUAD	RRMSE	0.329	0.292	0.278	0.271	0.292	0.354	0.313	0.298	0.289	0.314
	RBIAS	0.256	0.261	0.262	0.263	0.261	0.275	0.279	0.28	0.28	0.279
VAT	RRMSE	0.277	0.256	0.246	0.242	0.255	0.371	0.328	0.313	0.303	0.329
	RBIAS	0.233	0.237	0.237	0.237	0.236	0.289	0.292	0.294	0.294	0.292
				Rice 4					Rice 5		
Estimator		10	25	50	100	Mean	10	25	50	100	Mean
AO2Q	RRMSE	0.318	0.181	0.123	0.092	0.178	0.221	0.134	0.105	0.091	0.138
	RBIAS	0.142	0.075	0.057	0.05	0.081	0.004	-0.047	-0.06	-0.067	-0.043
AO3Q	RRMSE	0.31	0.222	0.19	0.172	0.224	0.265	0.164	0.117	0.089	0.159
	RBIAS	-0.119	-0.143	-0.151	-0.152	-0.141	-0.021	-0.034	-0.034	-0.036	-0.031
BDAV3	RRMSE	0.45	0.341	0.291	0.262	0.336	0.283	0.177	0.125	0.088	0.168
	RBIAS	0.226	0.228	0.231	0.23	0.229	-0.01	-0.016	-0.015	-0.016	-0.014
KM2P	RRMSE	0.415	0.299	0.254	0.224	0.298	0.262	0.172	0.128	0.093	0.164
	RBIAS	0.191	0.189	0.194	0.192	0.192	0.037	0.043	0.049	0.043	0.043
KMP	RRMSE	0.252	0.147	0.101	0.074	0.143	0.261	0.161	0.113	0.08	0.154
	RBIAS	0.001	-0.015	-0.017	-0.021	-0.013	0.036	0.024	0.018	0.014	0.023
OD2C	RRMSE	0.33	0.23	0.197	0.178	0.234	0.277	0.168	0.121	0.084	0.163
	RBIAS	-0.118	-0.142	-0.153	-0.155	-0.142	0.029	0.013	0.014	0.012	0.017
OD3C	RRMSE	0.284	0.186	0.15	0.129	0.187	0.222	0.134	0.096	0.068	0.13
	RBIAS	-0.07	-0.09	-0.098	-0.101	-0.09	0.008	-0.003	-0.004	-0.004	-0.001
QUAD	RRMSE	0.483	0.427	0.41	0.397	0.429	0.3	0.279	0.272	0.269	0.28
	RBIAS	0.387	0.385	0.388	0.386	0.387	0.261	0.262	0.263	0.265	0.263
VAT	RRMSE	0.421	0.385	0.373	0.366	0.386	0.269	0.248	0.24	0.238	0.249
	RBIAS	0.357	0.357	0.359	0.359	0.358	0.233	0.233	0.233	0.234	0.233

Table 3: Complete results for all estimators and data sets continued

				Waterfowl					Xanth		
Estimator		10	25	50	100	Mean	10	25	50	100	Mean
AO2Q	RRMSE	0.36	0.359	0.363	0.366	0.362	0.256	0.158	0.128	0.113	0.164
	RBIAS	-0.297	-0.341	-0.355	-0.362	-0.339	-0.004	-0.065	-0.082	-0.091	-0.06
AO3Q	RRMSE	0.564	0.565	0.564	0.565	0.565	0.288	0.218	0.195	0.181	0.221
	RBIAS	-0.533	-0.555	-0.559	-0.563	-0.552	-0.146	-0.156	-0.164	-0.166	-0.158
BDAV3	RRMSE	0.361	0.256	0.206	0.179	0.251	0.412	0.272	0.181	0.133	0.249
	RBIAS	-0.151	-0.143	-0.142	-0.144	-0.145	0.02	0.018	0.013	0.019	0.017
KM2P	RRMSE	0.368	0.233	0.163	0.117	0.22	0.421	0.272	0.198	0.144	0.259
	RBIAS	-0.017	-0.01	-0.014	-0.013	-0.013	-0.046	-0.055	-0.052	-0.052	-0.051
KMP	RRMSE	0.664	0.674	0.679	0.68	0.674	0.26	0.211	0.192	0.182	0.211
	RBIAS	-0.648	-0.669	-0.677	-0.679	-0.668	-0.147	-0.167	-0.169	-0.17	-0.163
OD2C	RRMSE	0.586	0.587	0.593	0.594	0.59	0.311	0.245	0.227	0.217	0.25
	RBIAS	-0.551	-0.577	-0.588	-0.591	-0.577	-0.184	-0.195	-0.202	-0.204	-0.196
OD3C	RRMSE	0.546	0.547	0.552	0.553	0.55	0.266	0.213	0.196	0.187	0.215
	RBIAS	-0.514	-0.537	-0.548	-0.551	-0.538	-0.159	-0.169	-0.174	-0.175	-0.169
QUAD	RRMSE	0.55	0.389	0.34	0.306	0.396	0.173	0.115	0.088	0.072	0.112
	RBIAS	0.277	0.262	0.271	0.269	0.27	-0.048	-0.05	-0.051	-0.05	-0.05
VAT	RRMSE	0.55	0.416	0.374	0.345	0.421	0.142	0.098	0.076	0.063	0.095
	RBIAS	0.324	0.313	0.317	0.316	0.318	-0.047	-0.048	-0.047	-0.047	-0.047
				Bee eater					PG 92		
Estimator		10	25	50	100	Mean	10	25	50	100	Mean
AO2Q	RRMSE	0.2	0.167	0.157	0.153	0.169	0.224	0.211	0.207	0.206	0.212
	RBIAS	-0.108	-0.133	-0.142	-0.146	-0.132	-0.167	-0.192	-0.198	-0.202	-0.19
AO3Q	RRMSE	0.185	0.136	0.113	0.101	0.134	0.123	0.08	0.058	0.045	0.076
	RBIAS	-0.11	-0.095	-0.092	-0.089	-0.097	-0.062	-0.041	-0.033	-0.029	-0.041
BDAV3	RRMSE	0.325	0.315	0.311	0.309	0.315	0.404	0.399	0.398	0.397	0.4
	RBIAS	-0.308	-0.307	-0.308	-0.307	-0.307	-0.397	-0.395	-0.396	-0.397	-0.396
KM2P	RRMSE	1.239	0.776	0.548	0.393	0.739	0.606	0.392	0.281	0.207	0.371
	RBIAS	-0.045	-0.058	-0.065	-0.058	-0.057	-0.072	-0.077	-0.08	-0.081	-0.077
KMP	RRMSE	0.171	0.132	0.116	0.108	0.132	0.122	0.081	0.059	0.047	0.077
	RBIAS	0.081	0.09	0.094	0.097	0.09	0.006	0.018	0.022	0.024	0.017
OD2C	RRMSE	0.201	0.147	0.127	0.114	0.147	0.2	0.159	0.143	0.136	0.16
	RBIAS	0.078	0.092	0.098	0.099	0.092	0.096	0.115	0.12	0.124	0.114
OD3C	RRMSE	0.154	0.107	0.089	0.076	0.106	0.119	0.077	0.06	0.048	0.076
	RBIAS	0.05	0.058	0.062	0.062	0.058	0.009	0.023	0.027	0.029	0.022
QUAD	RRMSE	0.094	0.061	0.043	0.033	0.058	0.077	0.052	0.041	0.034	0.051
	RBIAS	0.016	0.017	0.016	0.017	0.017	0.026	0.025	0.026	0.026	0.026
VAT	RRMSE	0.066	0.043	0.032	0.025	0.042	0.048	0.031	0.023	0.018	0.03
	RBIAS	-0.014	-0.014	-0.014	-0.014	-0.014	-0.008	-0.01	-0.01	-0.01	-0.009
				PG 93							
Estimator		10	25	50	100	Mean					
AO2Q	RRMSE	0.213	0.189	0.183	0.182	0.192					
	RBIAS	-0.144	-0.165	-0.172	-0.176	-0.164					
AO3Q	RRMSE	0.17	0.124	0.105	0.095	0.123					
	RBIAS	-0.118	-0.096	-0.089	-0.087	-0.098					
BDAV3	RRMSE	0.324	0.318	0.316	0.315	0.318					
	RBIAS	-0.316	-0.315	-0.314	-0.315	-0.315					
KM2P	RRMSE	0.645	0.424	0.319	0.249	0.409					
	RBIAS	-0.145	-0.158	-0.15	-0.152	-0.151					
KMP	RRMSE	0.155	0.099	0.075	0.057	0.097					
	RBIAS	-0.043	-0.037	-0.035	-0.033	-0.037					
OD2C	RRMSE	0.194	0.118	0.083	0.059	0.114					
	RBIAS	-0.019	-0.01	-0.011	-0.009	-0.012					
OD3C	RRMSE	0.155	0.1	0.075	0.06	0.098					
	RBIAS	-0.048	-0.04	-0.04	-0.04	-0.042					
QUAD	RRMSE	0.12	0.077	0.055	0.042	0.073					
	RBIAS	0.018	0.021	0.019	0.019	0.019					
VAT	RRMSE	0.077	0.049	0.033	0.024	0.046					
	RBIAS	0.003	0.004	0.004	0.003	0.004					
